# Growth Factors as Treatment for Inflammatory Bowel Disease: A Concise Review of the Evidence Toward Their Potential Clinical Utility

**DOI:** 10.4103/1319-3767.54742

**Published:** 2009-07

**Authors:** Josué Barahona-Garrido, Jorge Hernández-Calleros, Ignacio García-Juárez, Jesús K. Yamamoto-Furusho

**Affiliations:** 1 Inflammatory Bowel Disease Clinic, Department of Gastroenterology, Instituto Nacional de Ciencias Médicas y Nutrición Salvador Zubirán, Mexico City, Guatemala; 2 Universidad Nacional Autónoma de México, Mexico City, Mexico, Guatemala; 3 Instituto de Enfermedades Digestivas y Nutricionales, Guatemala City, Guatemala

**Keywords:** Inflammatory bowel disease, growth factors, Crohn's disease, growth hormone, colony-stimulating factors

## Abstract

In the process of inflammation and repair of the intestinal mucosa in inflammatory bowel disease (IBD), there occurs a complex and an unknown interplay of innate and adaptive immune mechanisms. This interaction of factors may explain why IBD is characterized by a relapsing and remitting clinical course. Different components of innate immunity, hormones and interleukins in IBD have been suggested to be impaired. The growth hormone, epidermal growth factor, keratinocyte growth factor and colony-stimulating factors have emerged as potential tools for the modulation of intestinal inflammation and repair. Despite promising results of initial studies, the evidence that justify treatment of patients in clinical practice is not enough as some of the trials are nonrandomized or included a small number of patients. In this concise review, we provide a summary of the most recent and relevant evidence regarding the potential therapeutic effects of growth factors in IBD.

Crohn's disease (CD) and ulcerative colitis (UC) represent a group of diseases designated as inflammatory bowel disease (IBD). These chronic disorders characterized by inflammation of the gastrointestinal tract have a relapsing and remitting clinical course. IBD is thought to be a multifactorial disease as distinct mechanisms have been proposed.[1] There is evidence that the pathogenesis of IBD includes a complex, and not clearly defined, interplay between immune disorders and imbalance of inflammatory mediators and hormones, which lead to intestinal mucosal damage and defective repair. Growth factors have emerged as potential tools for modulation of intestinal inflammation and repair, having an important role in cellular proliferation, differentiation and angiogenesis. At least 30 different growth factors are relevant in maintaining gut mucosal integrity. The evidence of utility of growth hormone (GH), granulocyte-monocyte colony-stimulating factor (GM-CSF) and granulocyte CSF (G-CSF) comes from patients with CD; a majority of trials are open-labeled, nonrandomized and with a small number of patients. Epidermal growth factor (EGF) and keratinocyte growth factor (KGF) have been studied mostly in patients with UC, providing evidence that both GFs play a role in the healing of damaged colonic tissue. It seems that new insights into reparative components of mucosal homeostasis may provide effective synergic or single-agent treatment alternatives to immunosuppression for IBD.[2] Despite promising results, we need more evidence to recommend them as a therapy in clinical daily practice.[3]

## GROWTH HORMONE

There is evidence that GH enhances survival, remission of inflammation and mucosal repair in dextran sodium sulfate–induced colitis in transgenic mice that overexpress GH.[4]

Young patients with Crohn's disease and growth impairment, despite a normal stimulation and spontaneous secretion of GH, have a reduced plasmatic concentration of insulin type-1 growth factor (IGF-1), which suggests resistance to GH.[5] The imbalance between GH and IGF-1 may be explained in part by the action of interleukin-6 (IL-6).[6]

A double-blind and placebo-controlled study in 37 patients with moderate-to-severe active Crohn's disease (CD) showed that therapy with recombinant GH (Somatropin, Humatrope, Eli Lilly, Indianapolis, USA) may be a beneficial treatment. In this trial, the GH dose of 5 mg/day subcutaneously for 1 week, followed by 1.5 mg/day maintenance dose for 4 months was superior to placebo with regard to reducing Crohn's disease activity index (CDAI)[7] by a mean of 143 ± 144 and 19 ± 63, respectively.[8]

GH and IGF-1 are relevant for IBD because of their trophic effects on epithelial cells, mesenchymal cells and intestinal immune cells. Even though it appears that GH therapy is beneficial, potential risks and complications by its direct effects or induction of IGF-1 are possible. It is suggested that IGF-1 can produce an increased risk of intestinal cancer and fibrosis.[9] GH have shown a potential carcinogenic role in colorectal cancer,[10] primary Ki-1 lymphoma of the skin,[11] liposarcoma and lipoma,[12] hepatocarcinoma,[13] breast cancer,[14] prostate cancer[15] and uterine cervical cancer.[16] The trials regarding the usefulness of GH in IBD have not reported an increased incidence of cancer.

## EPIDERMAL GROWTH FACTOR

Human EGF is a 53-aminoacid peptide produced by the salivary and Brunner's duodenal glands. It is a powerful mitogen and it seems to play a key role in the healing response of the gastrointestinal tract.[17] The recognition that various important growth factors, the EGF among them, regulate and maintain the barrier function of colonic mucosa, decrease mucosal permeability and promote tissue repair stimulated the investigation of the therapeutic role of growth factors in inflammatory bowel disease.[18]

In a randomized double-blind placebo-controlled trial, Sinha *et al.* studied 24 patients with mild-to-moderate left-sided UC. Patients were randomized to receive either daily enemas of 5 *μ*g of EGF in 100 mL of an inert carrier or enemas with carrier alone for 14 days. All patients received oral mesalamine daily. Primary endpoint was disease remission after two weeks of treatment defined by a St. Marks score ≤ 4 and absence of inflammation in sigmoidoscopy. After 2 weeks, 10 of 12 patients treated with EGF enemas were in remission, whereas 1 of 12 in the control group (83% vs 8%, *P* < 0.001) was in remission. At the second-week assessment, endoscopic score, histological score and disease activity score were also better in the EGF group than in the placebo group (*P* < 0.01).[19] These excellent results encourage further trials with a greater number of patients in order to confirm results and determine the optimal dose, route of administration and duration of therapy. The potential carcinogenic role of EGF therapy in the intestine remains to be addressed because EGF may be implicated in tumorigenesis as well as in targeted treatment for colorectal cancer,[20,21] esophageal cancer,[22] head and neck cancer,[23] hepatocellular carcinoma,[24] pancreatic cancer,[25] non-small cell lung cancer,[26,27] malignant glioma[28] and breast cancer,[29] among others. No cases of colorectal cancer have been reported after EGF therapy for IBD, probably because of the small number of patients included in trials.

## KERATINOCYTE GROWTH FACTOR

The fact that keratinocyte growth factor (KGF) enhances tissue repair of the skin leads to investigate its effect on gastrointestinal tract. The KGF expression was found to be increased and correlated with the degree of histological inflammation and interleukin-1 beta in IBD patients, suggesting that KGF has a role in the epithelial repair, especially in patients with UC.[30,31] Initial evidence was obtained from 2,4,6-trinitrobenzenesulfonic acid/ethanol-induced colitis and from dextran sodium sulfate–induced colitis mice models in whom intraperitoneal KGF-1 (also known as fibroblast growth factor-7) and KGF-2 (also known as fibroblast growth factor-10) were showed to reduce macroscopic and microscopic lesions in colonic tissue.[32–34]

Sandborn *et al.* performed the only randomized, double-blind, placebo-controlled trial of the use of KGF-2 (Repifermin; Human Genome Sciences Inc., Rockville, Maryland, USA). In this trial, 88 mild-to-moderate active and refractory-to-treatment patients were randomized to receive 1, 5, 10, 25 or 50 *μ*g/kg, or placebo intravenously for 5 consecutive days. Despite well tolerance of KGF-2 treatment, after 6 weeks there was no evidence that it was superior to placebo in endoscopic, histopathological and quality-of-life scores.[35] There are no studies with higher dosages or longer treatments with KGF-2. Furthermore, carcinogenic effect of KGF-2 therapy in patients with IBD remains to be studied.

## COLONY-STIMULATING FACTORS

Different components of innate immunity in IBD have been suggested to be impaired,[1,36] similar to those in a wide variety of IBD-related diseases that respond to therapy with CSF.[37,38] Some defects of innate immunity in IBD patients that have been described are as follows: impaired migration of neutrophils,[39] complement dysfunction,[40] decreased phagocytic and bactericidal neutrophil function,[41] deficient superoxide generation in neutrophils,[42] diminished production of IL-8 and IL-1β from macrophages[43] and production of large amounts of proinflammatory cytokines, such as IL-23, tumor necrosis factor-alpha, IL-6 and IL-17 in certain macrophages.[44–47] Furthermore, there is evidence that patients with CD possess a weak innate inflammatory response,[48] with insufficient recruitment of neutrophils and inadequate removal of bacteria and other debris.[49]

GM-CSF has shown to ameliorate clinical and histological activity in induced-colitis animal models.[50] Furthermore, G-CSF showed in induced-immune complex colitis in White New Zealand rabbits that it can reduce proinflammatory mediators.[51] It seems that the onset of induced colitis in mice can be prevented,[52] and also polymorphonuclear apoptosis may be delayed[53] and the neutrophil tissue migration may be increased.[54] Recently, treatment with G-CSF showed to ameliorate murine dextran sulfate sodium–induced colitis by suppressing mucosal inflammation and epithelial damage in the rectum.[55] All these effects on neutrophils, proinflammatory mediators and epithelial cells may explain the potential therapeutic role of CSF.

There are a few of studies that support the therapy with CSF in IBD. All clinical studies have been performed in patients with CD and have several limitations. G-CSF has been studied only in open trials, and GM-CSF is the only CSF studied in a randomized trial.

### Granulocyte colony-stimulating factor

A pilot study with filgrastim (Neupogen; Amgen Inc, Thousand Oaks, California, USA) in five patients with clinically inactive CD (CDAI < 150) and severe endoscopic postoperative recurrence was performed, with the primary objective of evaluating safety and efficacy. Patients received 300 *μ*g of filgrastim subcutaneously, thrice a week, for 12 weeks. After treatment, complete mucosal healing occurred only in two patients, and closure of perianal fistulae was noted in one patient, suggesting that filgrastim seems to be safe, well tolerated, and might provide efficacy in CD.[56]

More recently, an open-labeled trial was conducted in 20 CD patients, with daily filgrastim dose of 300 *μ*g subcutaneously, for 12 weeks. All patients had a CDAI between 200 and 450. Five patients achieved remission (CDAI < 150) during the study, 11 showed a decrease of at least 70 points, and 3 of 4 demonstrated a closure of more than 50% of fistulae. At week 12, 4 of 11 responders maintained response for additional 4 weeks.[57]

### Granulocyte-monocyte colony-stimulating factor

Sargramostim (Immunex Corporation, Seattle, Washington, USA) was studied in an 8-week, open labeled, dose-escalating study on 15 patients with CD (475>CDAI>220). This study showed that 80% achieved clinical response (decrease of CDAI in 70) and 53% achieved remission (CDAI < 150). Dosages of 4, 6 and 8 *μ*g/kg/day were studied, and the response rates were found to be 75%, 85% and 75%, respectively. Furthermore, treatment also improved quality of life. Complete closure of a chronic rectovaginal fistula was observed in the only patient with this clinical feature.[58]

Recently, a multicenter, randomized, placebo-controlled trial included 124 patients randomly assigned in a 2:1 ratio to receive sargramostim (6 *μ*g/kg/day) or placebo subcutaneously. In this trial, the primary end point was not proven, as clinical response was noted in 54% in the sargramostim group, and 44% in the placebo group (*P* = 0.28). A decrease from baseline of at least 100 points in the CDAI score was higher in the treated than in the placebo group (48% vs 26%, *P* = 0.01). The remission rate was also superior (40% vs 19%, *P* = 0.01). The superiority of sargramostim was also noted 30 days after treatment. Fistulae were eliminated in four of eight patients in the treated group, and in two of five in the placebo group.[59]

## CONCLUSION

(1) There is evidence suggesting that IBD may result from alteration in the balance between some hormones and cytokines involved in the regulation of intestinal cell homeostasis. These findings lead to investigate the potential effect of administering GH and EGF to patients with CD and UC, finding excellent results in small sample trials. Despite there not being enough evidence regarding the potential carcinogenic effect of EGF in the intestine, it is suggested that EGF takes part in several tumors. KGF was initially studied in animal models and then in few patients with UC without demonstrating that is superior to placebo. (2) The hypothesis that CD may result from an impaired neutrophil migration, recruitment and function, lead to study CSF. Most of the evidence of the utility of CSF in CD comes from studies that are open-labeled, non-randomized and that included a limited number of patients. The only randomized and placebo-controlled study regarding the utility of GM-CSF showed promising results on reducing CDAI score, achieving remission and reducing fistulae. (3) With some of these therapies the question of the ideal duration, dosage, route of administration and adverse effects is still unknown. Moreover, clinicians require more information regarding its benefit as coadjuvant to conventional therapy for IBD, as well as quality of life and cost-effectiveness. Finally, we need more evidence to recommend GF in the daily clinical practice as their real efficacy has not yet been established; therefore, further studies are warranted.
